# Interpreting the actionable clinical role of rare variants associated with short QT syndrome

**DOI:** 10.1007/s00439-024-02713-x

**Published:** 2024-11-06

**Authors:** Estefanía Martínez-Barrios, Andrea Greco, José Cruzalegui, Sergi Cesar, Nuria Díez-Escuté, Patricia Cerralbo, Fredy Chipa, Irene Zschaeck, Leonel Slanovic, Alipio Mangas, Rocío Toro, Josep Brugada, Georgia Sarquella-Brugada, Oscar Campuzano

**Affiliations:** 1 Arrhythmias, Inherited Cardiac Diseases and Sudden Death Unit, Hospital Sant Joan de Déu, Esplugues de Llobregat, 08950 Spain; 2 Arrítmies Pediàtriques, Cardiologia Genètica i Mort Sobtada, Malalties Cardiovasculars en el Desenvolupament, Institut de Recerca Sant Joan de Déu (IRSJD), Esplugues de Llobregat, 08950 Spain; 3 Medical Science Department, School of Medicine, Universitat de Girona, C/ Emili Grahit 77, Girona, Catalonia 17003 Spain; 4 Medicine Department, School of Medicine, University of Cádiz, Cádiz, 11002 Spain; 5 Biomedical Research and Innovation Institute of Cadiz (INiBICA), Cádiz, 11009 Spain; 6 Internal Medicine Department, Puerta del Mar University Hospital, Cádiz, 11009 Spain; 7 Arrhythmias Unit, Hospital Clinic, University of Barcelona, Barcelona, 08036 Spain; 8 Centro Investigación Biomédica en Red-Cardiovascular (CIBERCV), Madrid, 28029 Spain; 9 Pediatrics Department, School of Medicine, Universitat de Barcelona, Barcelona, 08036 Spain; 10 Institut d’Investigació Biomèdiques de Girona (IDIBGI-CERCA), Salt, 17190 Spain

## Abstract

Genetic testing is recommended in the diagnosis of short QT syndrome. This rare inherited lethal entity is characterized by structural normal hearts with short QT intervals in the electrocardiogram. Few families diagnosed with this arrhythmogenic disease have been reported worldwide so far, impeding a comprehensive understanding of this syndrome. Unraveling the origin of the disease helps to the early identification of genetic carriers at risk. However, only rare variants with a definite deleterious role should be actionable in clinical practice. Our aim was to perform a comprehensive update and reinterpretation, according to the American College of Medical Genetics and Genomics recommendations of all rare variants currently associated with short QT syndrome. We identified 34 rare variants. Reanalysis showed that only nine variants played a deleterious role associated with a definite short QT syndrome phenotype. These variants were located in the four main genes: *KCNQ1*, *KCNH2*, *KCNJ2* or *SLC4A3*. Additional rare variants located in other genes were associated with other conditions with phenotypic shortened QT intervals, but not definite diagnosis of short QT syndrome. Periodically updating of rare variants, especially those previously classified as unknown, helps to clarify the role of rare variants and translate genetic data into clinical practice.

## Introduction

Genetic analysis is a key tool currently used in the diagnosis of rare inherited arrhythmogenic syndromes (IAS) (Wilde et al. 2022). An early and accurate genetic diagnosis can be crucial to the delivery of personalized care for a patient and to identify at-risk family members. The American College of Medical Genetics and Genomics (ACMG) published standards and guidelines for the interpretation of rare variants (Richards et al. 2015), helping to decipher which, if any, of the observed variants in a genetic test are causative. These recommendations describe a framework of several items, and each one includes different evidence of pathogenicity and benignity, enabling the classification of rare variants. However, the lack of data in a large part of rare variants impedes a definite classification, with data remaining ambiguous (variants of unknown significance, VUS). Nowadays, it is widely accepted that VUS should not be used in clinical decision-making (Wilde et al. 2022). Therefore, ACMG recommendations described for classifying variants are according to a series of criteria. This framework is performed according to available data at the moment of classification but variants may alter previous classification over time as new clinical and basic evidence arises, especially continuous improvement of population frequencies. This fact reinforces the periodic reanalysis/reinterpretation of variants in IAS between year 2–5, especially if previously classified as VUS (Campuzano et al. 2020; Sarquella-Brugada et al. 2022).

Short QT syndrome (SQTS) is a rare IAS characterized by a shortened QT interval (QTc ≤ 340ms), tall and peaked T waves in the electrocardiogram (ECG), particularly in the precordial leads, and poor rate adaptation of the QT interval; all without structural heart abnormalities (Wilde et al. 2022). Diagnosis could be also considered in the presence of a QTc ≤ 360ms and one or more of the following: (a) a confirmed deleterious variant, (b) a family history of SQTS, (c) a family history of SCD at age 40 years, (d) survival from a ventricular tachycardia/ventricular fibrillation episode in the absence of heart disease (Wilde et al. 2022). Symptoms may range from asymptomatic to sudden cardiac death (SCD) and are usually strongly linked to both atrial fibrillation (AF) and ventricular arrhythmias. In approximately 40% of cases, SCD is the first manifestation of this disease (Mazzanti et al. 2014). This malignant arrhythmia can occur at any time, although most cases occur at a young age (El-Battrawy et al. 2019). The prevalence of SQTS is estimated at less than 1/10,000, with a lowest male predominance, despite both prevalence as well as sex ratio possibly being an underestimate due to the lack of families with a conclusive diagnosis worldwide to date (Wilde et al. 2022). This absence of families with SQTS impedes a proper clinical risk stratification and exhaustive genetic research. To date, pathogenic/likely pathogenic variants (P/LP) have been reported in only four genes following an autosomal dominant pattern of inheritance (*KCNH2*, *KCNJ2*, *KCNQ1* and *SLC4A3*). A complete genetic analysis identifies the cause of the SQTS in nearly 30% of families (Wilde et al. 2022). Additional rare variants in other genes have been potentially associated with overlapping phenotypes and concomitant shorter than normal intervals despite no definite diagnosis of SQTS (Walsh et al. 2022). Familiar analysis is highly recommended if a patient is clinically diagnosed, as well as phenotype-genotype segregation/correlation, is an identified causative genetic variant identified that; it should be done in all relatives, despite asymptomatic patients, due to a genetic carrier that can be at risk of malignant arrhythmias (Wilde et al. 2022). For this reason, obtaining a definite role of a genetic variant is crucial. To date, no exhaustive update, including reanalysis and reclassification of all reported rare variants associated with SQTS and overlapping phenotypes, has been performed to clarify a definite gene-disease association and the implementation of more rigorous frameworks.

## Materials and methods

A comprehensive analysis of all available data on each rare variant associated with SQTS was performed independently by three of the authors (March, 2024). All complied data were compared and verified. It is important to highlight that we only analyzed articles including rare variants associated with definite or potential phenotypes of SQTS, not all available articles concerning SQTS. Finally, all authors critically revised and discussed the clinical and genetic data included in the article. No studies were removed from our analysis due to inaccurate or incomplete clinical diagnoses or genetic studies. Data were collected from the PubMed (https://pubmed.ncbi.nlm.nih.gov/), ClinVar (www.ncbi.nlm.nih.gov/clinvar/intro/), the National Center for Biotechnology Information single-nucleotide polymorphism (SNP) database (www.ncbi.nlm.nih.gov/SNP), Google Scholar (www.scholar.google.es), Index Copernicus (http://en.indexcopernicus.com), Springer Link (www.link.springer.com) and Science Direct (www.sciencedirect.com). All rare genetic variants identified were contrasted with MasterMind (https://mastermind.genomenon.com), LitVar2 (www.ncbi.nlm.nih.gov/research/litvar2/) and last version (v4) of Genome Aggregation Database -GnomAD- (http://gnomad.broadinstitute.org/) for global population frequencies.

Focusing on updating all available data, all rare variants identified were reclassified according to current ACMG standards and guidelines for the interpretation of sequence variants (ACMG classification) (Richards et al. 2015). The PM2 item in the ACMG classification was considered fulfilled if the minor allele frequency (MAF) in relevant population databases was ≤ 0.05% (Lek et al. 2016) due to SQTS is a rare disease (prevalence at least less than 1/2000, MAF:0,05%). Concerning the frequency of disease-causing variants, the vast majority of pathogenic variants are extremely rare (< 0,01%) (Kobayashi et al. 2017). Concerning PVS1, it should only be used for variants in genes where loss of function is a previously established disease mechanism (www.ncbi.nlm.nih.gov/projects/dbvar/clingen/) (Abou Tayoun et al. 2018). Other modifications, including suggested removal of the reputable source criteria PP5 and BP6 (Biesecker, Harrison, & ClinGen Sequence Variant Interpretation Working 2018), the use of the PP1/BS4 (co-segregation) and PP4 phenotype specificity criteria (Biesecker et al. 2024), and a new standard that converts a tool’s scores to PP3 and BP4 evidence strengths (Pejaver et al. 2022) were considered.

The final classification of a rare variant such as VUS may be due to a lack of data or incongruences in the available data (Arbustini et al. 2022; Arbustini et al. 2022). The current estimated prevalence for SQTS is less than 1/10,000 (MAF: <0,01%). Considering these items, we propose a subclassification of the VUS variants into three subgroups: VUS-LB, VUS-LP or VUS. The first step for this subclassification is to ensure the current definitive association of the gene with SQTS. The next step is to analyse the existence or not of data (if there is, it must be taken into account if this data is contradictory). Finally, the use of the MAF (prevalence of SQTS is the threshold), being very low (< 0.005% or no MAF), low (> 0.005% and < 0.01%), or medium (> 0.01%). We have already implemented this approach in previously published manuscripts focused on other IAS (Martinez-Barrios et al. 2023). All investigators discussed the data included in each item of the ACMG, and consensus was made in a final classification of all variants to avoid any bias.

## Results

**Fig. 1:**
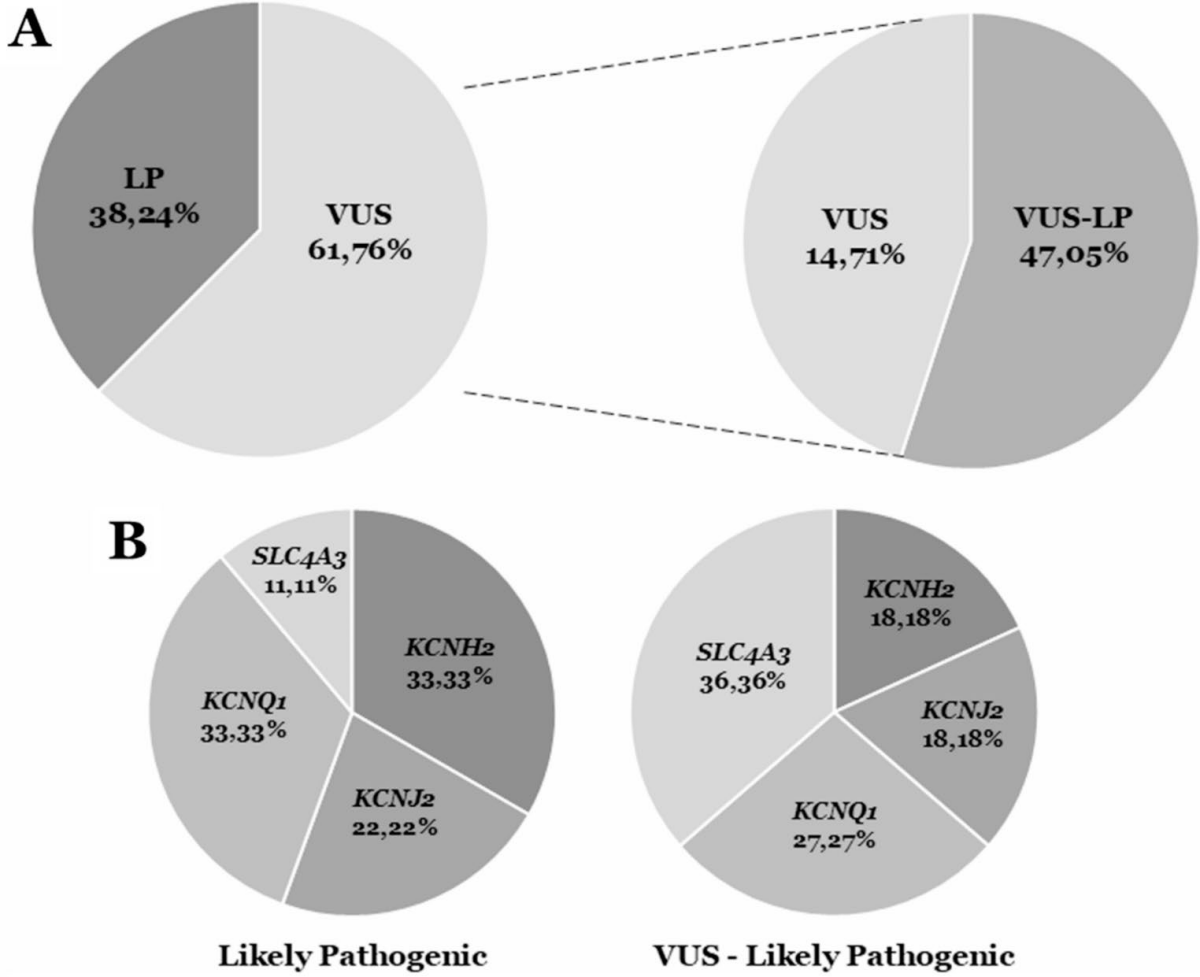
Reclassification of variants. **A.** Variants reclassified as Likely Pathogenic and remaining as unknown after update. Most part of variants of unknown significance should be considered as potentially damaging. **B.** Distribution in each gene of variants reclassified as likely pathogenic and with an unknown role but with a high potential deleterious role. LP: Likely Pathogenic. VUS: Variant of Unknown Significance. VUS-LP: Variant of Unknown Significance with a high potential damaging role

To date, current data concerning SQTS and overlapping phenotypes included a total of 34 rare variants (31 missense and four radical -one indel and three nonsense-) located in seven different genes (*CACNA1C*, *CACNB2*, *KCNH2*, *KCNJ2*, *KCNQ1*, *SLC22A5* and *SLC4A3*). Reinterpretation classified 13 as LP (38.24%) and 21 as VUS (61.76%). In this last group of VUS, our algorithm identified 15 (44.11%) with a high potential deleterious role (VUS-LP) and six (17.65%) remaining as VUS. Focusing only on patients with a definite diagnose of SQTS, we identified 20 rare variants (all missense) located only in four genes with a well-stablished association with SQTS (*KCNH2*, *KCNJ2*, *KCNQ1* and *SLC4A3*). Reinterpretation classified nine as LP (45%) and 11 as VUS (55%). In this last group of VUS, our algorithm identified all with a highly potential deleterious role (VUS-LP) (Tables 1 and 2; Fig. 1).

**Table 1:** Rare variants associated or potentially associated with short QT syndrome or overlapping phenotypes

Gene	Protein	Nucleotide	dbSNP	gnomAD	ClinVar	Phenotype	ACMG	Approach
*CACNA1C*	p.(Ala39Val)	c.116 C > T	rs121912776	NA	P	BrS + stnQT	VUS	VUS-LP
	p.(Gly490Arg)	c.1468G > C	rs121912775	NA	NA	BrS + stnQT	VUS	VUS-LP
	p.(Lys800Thr)	c.2399 A > C	rs369268832	0.0003%	VUS	ASD + stnQT	VUS	VUS
	p.(Arg1973Pro)	c.5918G > C	NA	NA	NA	BrS + stnQT	VUS	VUS-LP
*CACNB2*	p.(Ser480Leu)	c.1439 C > T	rs121917812	0.0003%	VUS	BrS + stnQT	LP	LP
*KCNH2*	p.(Glu50Asp)	c.150G > C	rs199472841	NA	NA	SQTS	VUS	VUS-LP
	p.(Thr152Ile)	c.455 C > T	rs794728354	0.0001%	VUS	BrS + stnQT	VUS	VUS
	p.(Arg164Cys)	c.490 C > T	rs1311655951	0.00007%	NA	BrS + stnQT	VUS	VUS
	p.(Ile560Thr)	c.1679T > C	NA	NA	NA	SQTS	LP	LP
	p.(Asn588Lys)	c.1764 C > G	rs104894021	NA	P	SQTS	LP	LP
	p.(Thr618Ile)	c.1853 C > T	rs199472947	NA	P	SQTS	LP	LP
	p.(Ser631Ala)	c.1891T > G	rs199472959	NA	NA	SQTS	VUS	VUS-LP
	p.(Trp927Gly)	c.2779T > G	NA	NA	NA	BrS + stnQT	VUS	VUS
	p.(Arg1135His)	c.3404G > A	rs199473547	0.0003%	VUS	BrS + stnQT	VUS	VUS
*KCNJ2*	p.(Asp172Asn)	c.514G > A	rs104894584	NA	P	SQTS	LP	LP
	p.(Glu299Val)	c.896 A > T	rs786205817	NA	LP	AF + SQTS	LP	LP
	p.(Met301Lys)	c.902T > A	NA	NA	NA	AF + SQTS	VUS	VUS-LP
	p.(Lys346Thr)	c.1037 A > C	NA	NA	NA	ASD + SQTS	VUS	VUS-LP
*KCNQ1*	p.(Val141Met)	c.421G > A	rs199472687	NA	P	AF + SQTS	LP	LP
	p.(Arg259His)	c.776G > A	rs199472720	0.001%	VUS	AF + SQTS	VUS	VUS-LP
	p.(Phe279Ile)	c.835T > A	rs1057519584	0.0002%	P	SQTS	LP	LP
	p.(Ala287Thr)	c.859G > A	rs765665086	0.002%	VUS	SQTS	VUS	VUS-LP
	p.(Phe299Val)	c.1435T > G	NA	NA	NA	AF + SQTS	VUS	VUS-LP
	p.(Val307Leu)	c.919G > C	rs120074195	NA	P	AF + SQTS	LP	LP
*SLC22A5*	p.(Phe17Leu)	c.51 C > G	rs11568520	0.002%	P	RCTD + stnQT	LP	LP
	p.(Phe23del)	c.64delTTC	rs377767444	0.001%	P	RCTD + stnQT	LP	LP
	p.(Trp62Ter)	c.185G > A	rs1554085942	NA	LP	RCTD + stnQT	VUS	VUS-LP
	p.(Arg289Ter)	c.865 C > T	rs386134212	0.0005%	P	RCTD + stnQT	LP	LP
	p.(Arg471Ter)	c.1411_1413delCGCinsTGA	NA	NA	NA	RCTD + stnQT	VUS	VUS-LP
*SLC4A3*	p.(Arg370His)	c.1109G > A	NA	0.0001%	P	SQTS	LP	LP
	p.(Arg600Cys)	c.1798 C > T	rs2106192912	NA	NA	SQTS	VUS	VUS-LP
	p.(Arg621Trp)	c.1861 C > T	rs764719743	0.0002%	NA	SQTS	VUS	VUS-LP
	p.(Glu852Asp)	c.2556G > T	NA	NA	NA	SQTS	VUS	VUS-LP
	p.(Arg952His)	c.2855G > A	rs1553552538	0.0002%	VUS	SQTS	VUS	VUS-LP

AF: Atrial Fibrillation, ASD: Autism Spectrum Disorder, stnQT: shorter than normal QT, BrS: Brugada Syndrome, gnomAD: genome aggregation database, ACMG: American College of Medical Genetics and Genomics, ClinVar: clinical variation, LP: likely pathogenic, LQTS: long QT syndrome, NA: not available, P: pathogenic, RCTD: renal carnitine transport defect, SQTS: short QT syndrome, VUS: variant of Uncertain significance

**Table 2:** Classification of variants

	Variants	LP	VUS	
			VUS-LP	VUS
Total	34	13 (38.24%)	16 (47.05%)	5 (14.71%)
Main Genes	20	9 (45%)	11 (55%)	0 (0%)

Number and percentage of variants classified as likely pathogenic (LP), variants unknown significance (VUS) and VUS-LP. The first row refers to the total number of variants and the second row refers to the variants in the four main genes (*KCNQ1*, *KCNH2*, *KCNJ2* and *SLC4A3*) only associated with a definite SQTS phenotype

### The main four genes

**Fig. 2:**
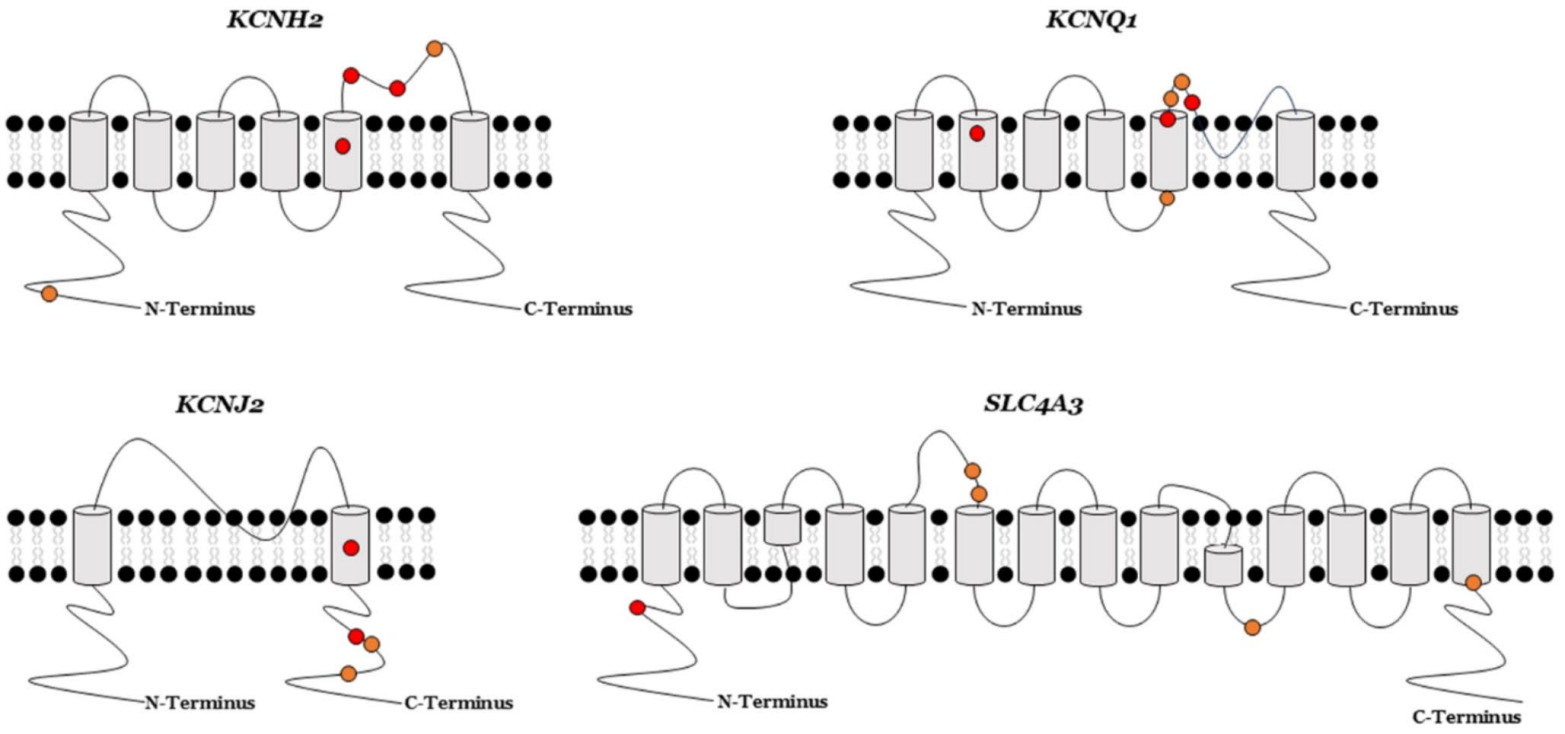
Localization of variants. Variants classified as likely pathogenic (red cercle) and classified as unknown but with a high potential deleterious role (orange cercle) in each of the four main genes (*KCNQ1*, *KCNH2*, *KCNJ2* and *SLC4A3*) associated with Short QT Syndrome

We identified a total of 24 rare variants located in four main genes (*KCNH2*, *KCNJ2*, *KCNQ1* and *SLC4A3*). Twenty of them were associated with a definite phenotype of SQTS: five in the *KCNH2* gene, four in the *KCNJ2* gene, six in the *KCNQ1* gene and five in *SLC4A3*. The four remaining variants were located in the *KCNH2* gene, but were associated with a phenotype of Brugada Syndrome (BrS) with a shorter than normal QT interval (stnQT). The main gene currently associated with SQTS is *KCNH2*, as observed in our study. We identified nine rare missense variants reported as potentially causative for phenotypes associated with SQTS. However, our comprehensive analysis showed that four out of nine variants (p.Thr152Ile, p.Arg164Cys, p.Trp927Gly and p.Arg1135His) were related to a phenotypic condition of BrS and stnQT, as also reported in other genes (*CACNA1C* and *CACNB2*) (please, see sections below). Therefore, only five rare variants have been reported to date in families diagnosed with a definite SQTS phenotype. Three of these were classified with a definite LP role (p.Ile560Thr, p.Asn588Lys and p.Thr618Ile) and two remaining as VUS (p.Glu50Asp and p.Ser631Ala), following the ACMG recommendations. Lack of data impedes a conclusive role of these two rare VUS. However, our approach suggested a highly deleterious role (VUS-LP) due to lack of available MAF in global population databases (Tables 1 and 2 ; Figs. 1 and 2). In the *KCNJ2* gene, a total of four rare missense variants were identified in cases with definite SQTS. Two of these rare variants (p.Asp172Asn and p.Glu299Val) were classified with a definite LP role following ACMG recommendations; the first variant was associated with SQTS and the second variant was related to AF plus SQTS. The two other variants (p.Met301Lys and p.Lys346Thr) were classified as VUS, following ACMG recommendations; the first variant was related to AF plus SQTS and the second variant was reported in one case of autism spectrum disorder (ASD) and SQTS. These two VUS showed no MAF to date and our approach considered both with a highly suspected deleterious role (VUS-LP) (Tables 1 and 2; Figs. 1 and 2). In the *KCNQ1* gene, six rare missense variants were identified, three with a definite LP role and three remaining as VUS following ACMG recommendations. The three LP variants were reported in patients with a definite diagnosis of SQTS (p.Phe279Ile) or in other patients with SQTS and concomitant AF (p.Val141Met, p.Val307Leu). Similarly, the three variants classified as VUS were identified in cases with definite SQTS (p.Ala287Thr) and SQTS with concomitant AF (p.Arg259His, p.Phe299Val). These last three VUS were reclassified with a highly suspected deleterious role (VUS-LP) following our approach due to very low or unavailable MAF so far (Tables 1 and 2; Figs. 1 and 2). Finally, five rare missense variants were identified in the last main gene, *SLC4A3*. All these variants were identified in cases with a definite SQTS but only one showed a conclusive LP role (p.Arg370His) following ACMG recommendations. The other four rare variants (p.Arg600Cys, p.Arg621Trp, p.Glu852Asp and p.Arg952His) remain with an unknown role due to lack of data. However, these four VUS were predicted to have a highly potential deleterious role in our approach (VUS-LP) due to very low or unavailable MAF (Tables 1 and 2; Figs. 1 and 2).

### Overlapping phenotype-like variants

Nowadays, four rare missense variants have been reported in the *CACNA1C* gene associated with phenotypes, showing a reduction in the QT interval (p.Ala39Val, p.Gly490Arg, p.Lys800Thr and p.Arg1973Pro). Concretely, three variants were reported in cases diagnosed with BrS and stnQT (p.Ala39Val, p.Gly490Arg and p.Arg1973Pro) and one additional rare variant was reported in a patient diagnosed with ASD and stnQT (p.Lys800Thr). All these rare variants are currently classified as VUS, following ACMG recommendations; our approach suggested a highly potential deleterious role (VUS-LP) in three of them (p.Ala39Val, p.Gly490Arg and p.Arg1973Pro) due to unavailable MAF to date (Tables 1 and 2). In the *CACNB2* gene, only one rare missense variant was reported (p.Ser480Leu) as the cause of stnQT overlapping with BrS. This rare variant has been reported in several cases and functionally analyzed. Therefore, it is classified as LP, following ACMG recommendations. Despite other variants previously reported in this gene associated with a similar phenotype, an update of the available data showed no more deleterious variants (Tables 1 and 2). Finally, five rare variants (one missense, one indel and three nonsense) were identified in the *SLC22A5* gene. All these variants were identified in cases with a definite renal carnitine transport defect (RCTD) and stnQT. Three variants remained as LP, following ACMG recommendations (p.Phe17Leu, p.Phe23del and p.Arg289Ter).The other two rare variants (p.Trp62Ter and p.Arg471Ter) remained with an unknown role due to lack of data. However, both VUS were predicted to play a highly potential deleterious role in our approach (VUS-LP) due to unavailable MAF (Tables 1 and 2).

## Discussion

Genetic testing is part of the clinical diagnosis in IAS, including SQTS or phenotype-like entities. However, only variants with a conclusive role should be actionable in clinical practice (Landstrom et al. 2023). One of the main challenges is a precise interpretation of rare variants, the remaining large part with an unknown role following current ACMG recommendations. In addition, current guidelines recommend that genetic diagnosis in patients with SQTS or any phenotype-like variants, should be restricted only to genes with a definite disease-association (Wilde et al. 2022). This restricted approach helps to avoid overinterpretation of rare variants found in genes with little evidence for disease causality. For this reason, at our point of view, the main step before performing a genetic test is to conclude a definite clinical diagnosis (Martinez-Barrios et al. 2022). In our study, we updated all rare variants previously reported as the cause of SQTS or any phenotype-like variant. We used the available tools, but with a particular evidence-based framework to perform an exhaustive reinterpretation. We identified 34 rare variants but only nine variants still played a deleterious role associated with a definite SQTS phenotype after our reanalysis. These variants were located in the four principal genes (*KCNQ1*, *KCNH2*, *KCNJ2* or *SLC4A3*) currently associated with SQTS (Walsh et al. 2022). Additional rare variants situated in other genes were associated with other conditions with phenotypic shortened QT intervals, but did not lead definite diagnosis of SQTS. It is mandatory to clarify the clinical burden of variants in SQTS, especially those previously classified as VUS, helping to conclude a definite role and therefore, solve the dilemma for clinical teams and reduce the ambiguity in families carrying many rare variants, even in currently well-established genes. In consequence, recontact to families should be mandatory if a reclassification of variant occurs, mainly if it results in a change in clinical actionability.

### Main genes

Nowadays, only rare variants located in four genes have been associated with a definite phenotype of SQTS (Walsh et al. 2022; Wilde et al. 2022), as observed in our study. Concretely, out of 24 rare variants previously associated with SQTS, our reanalysis observed that only 20 rare variants were related to a definite phenotype of SQTS. The four remaining variants were located in the *KCNH2* gene but were associated with a phenotype of BrS with stnQT. Therefore, the four rare variants in this gene remain classified as VUS (p.Thr152Ile, p.Arg164Cys, p.Trp927Gly and p.Arg1135His), according to current available data of BrS patients with shorter QTc (Wang et al. 2014). In total, nine variants remain currently classified as LP following ACMG recommendations after the update (*KCNH2*: p.Ile560Thr, p.Asn588Lys and p.Thr618Ile. *KCNJ2*: p.Asp172Asn and p.Glu299Val. *KCNQ1*: p.Arg259His, p.Ala287Thr and p.Phe299Val. *SLC4A3*: p.Arg370His). It is imperative to remark that despite a LP role reported, clinical translation should be done with caution, especially without conclusive familial segregation. Therefore, a personalized genetic interpretation is also mandatory in actionable variants.

The main gene currently associated with SQTS is *KCNH2*, as observed in our study. We identified five variants, three of which classified as LP (p.Ile560Thr, p.Asn588Lys and p.Thr618Ile) (Butler et al. 2019; Du et al. 2022; Huang et al. 2021; Shiti et al. 2023; Zhang et al. 2022; Zhao et al. 2019), and the p.Ser631Ala (Akdis et al. 2018), variants remain as a definite deleterious role following the ACMG recommendations due to lack of sufficient data. The clinical role of both these variants may be underestimated and may lead to confusion in clinical translation. For this reason, our approach focused on definite diagnosis, definite gene and no MAF concluded a highly potential deleterious role as the cause of SQTS. In the *KCNJ2* gene, a total of four rare missense variants were identified in cases with definite SQTS, one in relation with isolated SQTS (p.Asp172Asn) (Du et al. 2021), two in patients with SQTS concomitant to other entities such as AF (p.Glu299Val and p.Met301Lys) (Deo et al. 2013; Hasegawa et al. 2014), or ASD (p.Lys346Thr) (Ambrosini et al. 2014). The first two rare variants remain classified with a definite LP role following the ACMG recommendations but the last two remain as VUS due to the lack of data. None showed MAF, but with a definite diagnosis and located in a definite gene, suggesting a highly potential deleterious role, as predicted by our approach. In the *KCNQ1* gene, we identified six rare missense variants related to SQTS, two associated with isolated SQTS (p.Phe279Ile and p.Ala287Thr) (Schneider et al. 2021), and four concomitant to AF (p.Val141Met, p.Arg259His, p.Phe299Val and p.Val307Leu) (Hong et al. 2005; Mazzanti et al. 2014; Moreno-Manuel et al. 2024; Wu et al. 2015). Three showed a definite LP role and three remained as VUS, following the ACMG recommendations, but three remained as VUS due to the lack of data (p.Arg259His, p.Ala287Thr and p.Phe299Val). Our approach predicted a highly suspected deleterious role due to be presented in patients with a definite diagnosis, being in a definite gene and showing a very low or unavailable MAF. The fourth gene is *SLC4A3*, and we identified five rare missense variants associated with a definite SQTS (p.Arg370His, p.Arg600Cys, p.Arg621Trp, p.Glu852Asp and p.Arg952His) (Christiansen et al. 2023; Thorsen et al. 2017). Only the first variant is classified as LP, following the ACMG recommendations. The other variants remain as VUS due to lack of adequate data to conclude a deleterious role. Our approach, supported by a definite diagnosis, being in a definite gene, and having a very low or unavailable MAF, concluded a highly potential deleterious role as a cause of SQTS.

All updated deleterious variants located in definite genes associated with SQTS are related to a high risk of malignant arrhythmias. Therefore, there is an urgent need to identify compounds that allow us to adopt effective pharmacological and non-pharmacological therapies in diagnosed patients. However, pharmacological treatment must be personalized in the SQTS subtype, depending on the altered gene. In recent years, studies using the human induced pluripotent stem cells (hiPSCs) derived to cardiomyocytes (CMs) have allowed to identify appropriate drugs for SQTS, especially type 1 (due to pathogenic variants in the *KCNH2* gene) (El-Battrawy et al. 2018; Zhao et al. 2019). In addition, other approaches have been proposed to help in the study of the potential drug response in clinical practice of a novel compound identified using hiPSC-CMs. The most promising tool has been mathematical modeling to provide insight into the drug effects on mechanisms of shortening (Jaeger et al. 2019; Jaeger et al. 2020). This dual-component therapy, also known as SupRep, uses cloning into a single construct of a custom-designed short hairpin *KCNH2* RNA with an approximately 80% reduction of the mutated allele (deletion) and a *KCNH2* cDNA with the correct version of the “short hairpin RNA-immune” allele (substitution). This therapy has been shown to effectively correct APD and rescue the LQT2 and SQT1 disease phenotypes in patient-derived iPSC-CM (in vitro)(Bains et al. 2022). However, these gene therapies are still in pre-clinical phases, and they still need to be tested in humans.

### Genes associated with phenotype-like variants

Our update showed that 14 rare variants should be considered with a deleterious or potentially deleterious role in entities with a corrected QT interval shorter than normal but not definite SQTS. Four of these rare variants are located in the *KCNQ1* gene (as mentioned before). Other 10 rare variants are placed on three genes: four in *CACNA1C*, one in *CACNB2* and five in the *SLC22A5* gene. These three last genes have been potentially associated with similar phenotypes to SQTS, in concordance to current data (Walsh et al. 2022; Wilde et al. 2022), but never with a conclusive definite diagnosis of SQTS, at least to date. Of these rare variants, only four remain classified with a definite pathogenic role following ACMG recommendations: one in *CACNB2* (p.Ser480Leu) associated with BrS and stnQT (El-Battrawy et al. 2021; Zhong et al. 2022), and three in the *SLC22A5* gene (p.Phe17Leu, p.Phe23del and p.Arg289Ter) associated with RCTD and stnQT following an autosomal recessive pattern of inheritance (Roussel et al. 2016). As mentioned, despite these pathogenic variants being associated with SQTS phenotype-like entities (BrS + stnQT, ASD + stnQT and RCTD + stnQT), translation into clinical practice should be performed in a personalized way. If adoption of treatment is carried out, recent published data should be taken into account, recent published data but also family segregation, and, most importantly, clinical symptoms of the patient. All other rare variants remain as VUS due to lack of data after a comprehensive update, therefore not actionable following the current guidelines (Wilde et al. 2022). However, no available MAF suggest a high potential deleterious role in each of phenotype-like variant identified, and all variants should be re-analysed periodically and not discarded as a potential cause of the reported phenotype.

Finally, it is important to note that rare variants located in two genes previously associated with SQTS phenotype-like, should not be included in the potential cause of disease due to updated data suggesting a non-deleterious role, mainly due to a non-conclusive phenotype or a high increase of MAF (*SCN5A*_p.Arg689His and *CACNA2D1*_p.Ser755Thr). Our update agrees with recent results supporting the disputed association of these variants/genes with SQTS phenotype-like cases (Walsh et al. 2022). Other variants in additional genes have been proposed as the cause of potential alterations in the reduction of the QT interval (*ANK2*_pArg3634Asp, *PKP2*_p.Asp26Asn and *ABCC9*_p.Arg633Cys) (Treat et al. 2021), but some of them have a MAF extremely high to be consider a causative variant of SQTS, and others require further studies to conclude a definite role in the SQTS or phenotype-like entities. In addition, an in vivo study has suggested that the ion channel modulator nitric oxide synthase 1 adaptor protein (Nos1ap) overexpression causes targeted subcellular localization of Nos1 to the CaV1.2 with a subsequent decrease of the QT interval in transgenic mice (Jansch et al. 2023). However, no clinical studies have been published to date to conclude a definite association in humans.

Our interpretation has some limitations. Firstly, the limited number of families reported worldwide with a definite diagnosis of SQTS impedes conducting a large and significant study. Second, the lack of available information for some analyzed variants impeded a comprehensive reanalysis of these rare variants, especially those classified as VUS. It is important to note that additional data may be available in the next future that may change the current reanalysis performed in our study. Lastly, those who should perform the reanalysis and assume the economic cost are not analyzed in our study, despite being a key point from our point of view.

## Conclusions

In summary, SQTS is an extremely malignant arrhythmogenic cardiac channelopathy. Periodical reanalysis and reinterpretation of rare variants associated with SQTS should be performed in all clinically diagnosed families. This update is especially necessary if genetic testing identifies rare variants previously classified as having an unknown role. Clarifying the role of a VUS allows an actionable role, helping to discern potential deleterious from benign variants. Our study shows that 45% of all currently reported variants play a conclusive lethal role in families with a definite diagnosis of SQTS. All these rare deleterious variants were located in the four main genes (*KCNQ1*, *KCNH2*, *KCNJ2* and *SLC4A3*) currently related to SQTS. Reported rare variants in other genes should be interpreted with caution due to overlapping phenotypes concomitant to shorter than normal QT intervals but no definite clinical diagnosis of SQTS.

## Data Availability

No datasets were generated or analysed during the current study.
